# Behavioral Implementation and Compliance of Anti-Epidemic Policy in the COVID-19 Crisis

**DOI:** 10.3390/ijerph18073776

**Published:** 2021-04-04

**Authors:** Chengzhe Fu, Liao Liao, Weijun Huang

**Affiliations:** School of Politics and Public Administration, South China Normal University, Guangzhou 510006, China; fcz_45@163.com (C.F.); 20181332013@m.scnu.edu.cn (W.H.)

**Keywords:** anti-epidemic policy, policy implementation and compliance, policy instruments, behavior effects of policy

## Abstract

Different countries have introduced different urgent policies to control the spread of the novel coronavirus. The compliance behavior of these anti-epidemic policies has always been an important concern to governments, and its effects need to be tested. In recent years, many scholars have paid attention to the mechanism and intervention of policy compliance behavior, which helps to explain the mechanism of anti-epidemic compliance behavior, and to improve the effectiveness of anti-epidemic policy. Therefore, considering the characters of youth groups in the context of the novel coronavirus, this study takes campus anti-epidemic compliance behavior as the research topic, based on 680 effective samples of college students in China, in order to examine the effectiveness of these policies using an investigation experiment. This study revealed that the ‘Nudge’ policy instrument was the most effective way to guide individuals’ behavior during the coronavirus outbreak, the ‘Sermon’ instrument was the least recognized, and the ‘Whip’ instrument (a traditional and classical policy instrument) had its normal effect on individuals’ behavior. Additionally, it found that high accessibility in policy implementation results in more significant policy behavior. By taking the effects of different policy behaviors into consideration, governments may produce better and more effective policy implementation and compliance during the anti-epidemic period.

## 1. Introduction

Since the novel coronavirus outbreak, different countries have enacted different urgent policies to control its spread. However, the implementation of these policies differs from general policy implementations, and its effectiveness needs to be examined. For example, Wuhan city in China was quarantined from 23 January to 8 April 2020, and Jiangsu province in China mobilized community organizations and volunteers to establish a digital tracking platform to prevent community epidemic spread [1]. Australia followed the AHMPPI (Australian Health Management Plan for Pandemic Influenza), and adopted border control and self-isolation or social and physical distancing [2], while Norway adopted the repressive strategy and control strategy through economic policy [3], etc. Meanwhile, the herd immunity project announced by Britain led to Brazilian tragedy [4]. Besides this, the quarantine policy in urban China was well implemented, but it caused protests and demonstrations in the United States. However, the non-participation and non-cooperation in rural areas also affected the implementation of anti-epidemic policies in China [5]. Policy effectiveness is influenced by two interactive aspects: policy implementation and policy compliance. Generally, a policy’s effectiveness depends on how it is implemented by the executive body and how well its design complies with the executive body’s objective. Most existing research focused on policy implementation, paying little attention to the aspect of policy compliance, and staying in dichotomy [6,7].

Most research has addressed policy implementation from the perspective of the executive body or the implementation process [8,9,10,11], in which policy implementation is seen as a tension-generating force in society. Tensions are generated between and within four components of the implementing process: idealized policy, implementing organization, target group, and environmental factors [12,13,14]. In the 1970s, scholars in the field of public policy began to study of policy implementation. Pressman and Wildavsky [15] insisted that a top-down approach be used to study policy implementation practice. Peters [16] pointed out that Western countries must deal with hierarchies of authority among multiple levels of government in the implementation of public policies. In China, Rong [17] opined that the implementation of public policy is mainly initiated by the central government and imposed through a system of downward pressure. He [10] illustrated that financial supports are key to the success of public policy implementation in China.

More recently, studies have explored the effectiveness of public policies from a policy compliance perspective, particularly for policies on information systems security [18,19] and privacy in a network service context. Other scholars have adopted a policy compliance perspective in empirical studies discussing information protection policies [20,21], or examining the influence on policy compliance in terms of the recognition and actions of the persons involved in the policy [22,23]. Moreover, some scholars have also explored the factors which may influence policy compliance effectiveness, such as the transparency affecting the effectiveness of defaults throughout the experiments [24,25]. Other scholars in public administration currently draw on theories and methods from psychology and related fields, and point to research in public administration which could benefit from further integration. Furthermore, it could be a two-way street for psychologists who want to test the external validity of their theories in a political-administrative setting [26].

The research on policy compliance can be deepened by paying more attention to policy compliance behavior. Behavioral public policy theory, which applies behavioral science to the field of public policy, argues that it is more efficient to change public behavior than it is to change policy formulations. Thaler [27] developed the concept of ‘Nudge’ as an important policy tool in behavioral public policy. ‘Nudge’ refers to guiding people’s behavior to change in the desired direction by changing the structure of choice, without relying on obvious economic stimulus or administrative means, under the premise of maintaining individual freedom of choice through non-mandatory intervention. In addition to ‘Nudge’, scholars have also proposed ‘Sermon’ as a policy tool. Unlike ‘Nudge’, ‘Sermon’ focuses on improving individuals’ ability to make better decisions, rather than their behavior [28]. By intervening in their behavior cognition or decision-making processes, people can exercise their own power and make choices based on their own will [29]. Tummers [28] argued that, in order to better implement policy, governments should rely on people’s support to guide and change their behavior. Similarly, Bowers [30] proposed applying ‘behavioral insight’ to guide public policy. 

Since the novel coronavirus outbreak originated in China, China started to implement policies to control the spread of the virus earlier, and has had more time to develop them. Thus, the outbreak appears to be more steadily controlled in China, which provides us opportunities to explore policy implementation and compliance as regards the coronavirus issue. To that end, an ‘investigation experiment’ [31] approach was adopted. Our research will mainly focus on Chinese public universities’ policies during the coronavirus outbreak, as we consider the university environment to be quite close to that of the larger society, with people having different social backgrounds and following societal rules governing power and resources. At the same time, choosing the policies of public universities facilitates the investigation experiment and provides highly reliable data for understanding the research question.

The following section first describes policy implementation and compliance problems in China—particularly during the coronavirus outbreak from February to March—and details our research questions and design, before presenting the data collection and analysis methods. The effects of policy behavior and ways to improve the effectiveness of policy implementation will be explored in the next section. The paper concludes by discussing the study’s contributions to the literature and its practical implications for local policy makers.

## 2. Research Questions and Research Design

### 2.1. Background and Questions

Policy implementation in China has three main forms: top-down implementation, bottom-up implementation, and integrated implementation. Top-down implementation emphasizes the central government promoting a policy to local governments and supervising it operation [8,10,11]. Bottom-up implementations are often promoted by local government through negotiation and interaction, and feature political mobilization in the implementation process. The integrated model of implementation focuses on the dynamics among the different levels of governments, such as policy networks and public dialogue, and other factors that influence policy implementation [9]. Generally, however, Chinese policy implementation follows the top-down model, with the central government defining the process, and local governments following it. However, policy implementation can sometimes show ‘deviation’ and ‘adjustment’ due to the influence of interest groups or a lack of institutional rules, making the evaluation of the implementation more challenging.

Following the outbreak of the novel coronavirus, different countries implemented different policies, with different factors influencing the effectiveness of those policies’ implementation. In China, the main causes of ineffective implementation have been non-participation and non-cooperation, mainly in rural areas [5]. However, as there is heterogeneity in different countries’ epidemic policies, the people in those countries may have different reactions. Thus, exploring related policy implementation and compliance processes and identifying the main influencing factors will provide new perspectives for scholars, and suggestions to policy makers in their considerations of coronavirus policy implementation and compliance.

Since the novel coronavirus outbreak, many urgent policies have been promulgated. While time constraints and the uncertainty surrounding the virus’ spread make it difficult to examine the effectiveness of different policies in a scientific way, the affected countries or regions must still explore ways of creating more effective and efficient policy. To that end, we should examine the effectiveness of the policies implemented during the coronavirus spread period through the lens of behavioral public policy theory. Additionally, as existing policies often have different target groups, and because policy implementation varies from one society to another, we must ask what kinds of policy instruments will yield better policy implementation in a global pandemic: the whip instrument, the sermon instrument, or the nudge instrument? Will the degree of policy compliance influence the policy effectiveness?

### 2.2. Research Design

In February 2020, following the coronavirus (COVID-19) outbreak in 2019, the Education Department of L Provincial Government issued an urgent notice barring students from returning to university, in order to reduce the risk of transmission. Q university adopted different ways of implementing the provincial notice. In the experimental design of this paper, the independent variable is the preference for different instruments of university policies, while the accessibility degree is reflected in the different ways of implementing the provincial notice. A randomized controlled trial (RCT) was used to collect the questionnaire data. All of the questionnaires were completed by mainland Chinese citizens, and the questionnaire should ensure homogeneity in all of the variables. Two factors were applied in the research design, based on four policy instruments (whip, sermon, nudge, control group) and two accessibility degrees (high, low); thus, eight experimental conditions were formed in this experiment. The coefficient of the variable of the epidemic prevention effect was 0.61(Cronbach’s α), which shows a good reliability.

The differentiation of policy instruments reflects three different ways of promoting the notice:

- The Whip instrument admonishes students to follow the policy or face a consequence, i.e., announcing that the university will “punish those who do not follow policy by recording major demerit in the university, and those who seriously violate the policy shall be transferred to the public security for disposal according to law”.
- The Sermon instrument seeks to enhance policy influence through slogans, such as “anti-epidemic situation, prevention of transmission, less going out, no going back to university”.
- The Nudge instrument encourages students to participate in online anti-epidemic actions, such as posting “I stand for the prevention policy of university Q’ by promising not to go back to university before the situation is controlled”.

The two degrees of accessibility reflected in the questionnaire are as follows:

- High accessibility requires that each student be well-informed, by forcing each to acknowledge their acceptance of the policy via WeChat (WeChat is the most popular social network in China, with over 1 billion users, similar to WhatsApp in Western countries).
- Low accessibility only requires publishing notices, without any mandatory compliance requirement.

The experimental groups are defined in Table 1 shown as below. H stands for the group, and in the brackets are the abbreviation of the policy instrument and accessibility degree. One subject can only be randomly assigned to one of the groups. For example, if the subjects were randomly assigned to H1, they would be tested as having read the material of the group “whip and high level”, and would answer the questions.

In addition to the eight experimental groups, other independent variables were measured by the questionnaire, including mood instability, psychological pressure, estimation about COVID-19, off-line participation, donation during COVID-19, the level of updates about COVID-19, trust in the central government during the COVID-19 period, and trust in local government during the COVID-19 period. The coefficient of the variable of university epidemic prevention effect was 0.62 (Cronbach’s α), which shows a good reliability (see more details in Appendix A).

## 3. Data Collection and Analysis of Data

### 3.1. Data Collection

In this study, the Qualtrics (Qualtrics is a general supported survey system. It supports a wide range of question types, from simple questions to advanced ones. In addition, Qualtrics can automatically record the experimental behavior, such as the number of clicks, the length of the experiment and so on. Please refer to https://www.qualtrics.com, accessed on 20 May 2020.) online survey system was used to collect the questionnaire data. The system randomly assigned the subjects to different experimental scenarios for the experiments, recording the experimental process and results, such as the experiment duration, click times, and answer selection. The questionnaire was distributed from February to March 2020, and 890 samples were collected and imported into Stata 16.0 (StataCorp, College Station, TX, USA). After excluding non-university students and samples with extreme response times (less than three standard deviations), we collected 680 valid samples. Table 2 shows that the sample was relatively balanced in gender, and that the participants were mainly about 20 years old, university undergraduates, non-party members, and lived in cities and towns in Guangdong Province, China. Their average monthly income was about 1000 Yuan. The numbers of confirmed cases in the participants’ locations were not high and, in most cases, the local government had begun to implement epidemic prevention measures in middle to late January. Given the operational difficulty of probability sampling during an epidemic period, and given the research topic of this study, this sample can be considered an effective research sample.

Eight experimental groups were randomly assigned: (1) the control group with low accessibility (n = 84); (2) the control group with high accessibility (n = 84); (3) the whip group with low accessibility (n = 75); (4) the whip group with high accessibility (n = 83); (5) the sermon group with low accessibility (n = 87); (6) the sermon group with high accessibility (n = 88); (7) the nudge group with low accessibility (n = 87); and, (8) the nudge group with high accessibility (n = 92). There was no significant difference (*p* > 0.05) in the distribution of the above demographic characteristics and the experimental actions among the eight experimental groups, indicating that the experimental groups have no significant inter-group difference, and that the random assignment operation was ideal (refer to Appendix B for Balance Check).

### 3.2. Analysis of the Data

The effect of anti-epidemic policy on individuals’ behavior mainly depends on the effectiveness, the cost performance, and the support of the target group. We mainly measured the effectiveness of anti-epidemic measures (the current anti-epidemic measures are very effective) and whether they were implementable for individuals (the current anti-epidemic measures can be implemented to individuals). The cost performance was mainly evaluated based on comparing the degree to which people were willing to follow the policy (the people consciously followed the current anti-epidemic measures) with the social cost of the policy (the social cost of implementing the current anti-epidemic measures was very high). Target group support was measured by group support perception (the people are very supportive of current anti-epidemic measures). Thus, the descriptive statistics of all the variables concerned in this research design are shown in Table 3 as below:

In our experiment, the behavioral effect of the university’s anti-epidemic policy was a dependent variable. Behavior change include effective behavior changes and efficient behavior changes, which means that the behavior changes caused by policy emphasis to produce the intended behavior change and to use minimal resources. Thus, regarding behavior effect of the anti-epidemic policy of university, the dependent variables include the policy effectiveness (the policy compliance degree) and the cost performance degree (refer to Appendix C).

Therefore, the research design was based on the experimental materials, including the effectiveness of campus policy, the cost performance of the university, and the support of the student groups.

## 4. Analysis of the Effects of Policy Behavior

**Figure 1:**
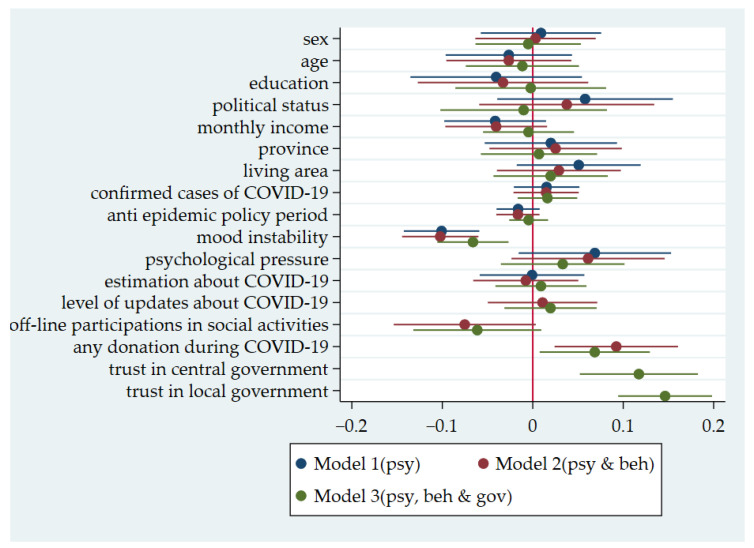
Effects of policy behavior.

In this study, multiple regression analysis was used to test the epidemic prevention effect, and the results are reported in Figure 1. We first took mood instability and psychological pressure (individual psychological dimensions) as independent variables, the effect of epidemic prevention policy behavior as dependent variables, and demographic variables as control variables for the regression analysis (Model 1). Mood instability had a significant negative effect on policy behavior effect, with a regression coefficient interval other than 0 (as below), while psychological pressure had a significant positive effect on policy behavior effect. Building on Model 1, Model 2 added epidemic prevention as an individual behavior variable; we found that only anti-epidemic donation behavior had a significant positive effect, with the model’s explanation rate increasing by only 1%, reaching 7%. Building on Model 2, Model 3 added trust in central and local governments, respectively, as variables. The regression results showed that trust in both the central and the local government had a significant positive effect on policy behavior, with the model’s interpretation rate reaching 19%, indicating that the effect of policy behavior depended mainly on the degree to which individuals trusted the government during the COVID-19 period (see Appendix D for details).

## 5. How to Improve the Effectiveness of Policy Implementation

Multiple regression analysis was used to test the university policy effectiveness, and the results were reported in Model 4, 5, and 6 in Table 4. Model 4 was used to explore the main effect of the instrument, which showed that, compared with the control group, the whip and nudge instruments reached significance, respectively (β = 0.16, *p* < 0.01; β = 0.13, *p* < 0.05). Similarly, we used Model 5 to investigate the main effect of the accessibility level, and it showed that, compared with low accessibility, high accessibility reached significance (β = 0.12, *p* < 0.01). Furthermore, Model 6 focused on the interaction effect, that is, the effect of the accessibility degree on different policy instruments. We found that, compared with the control group and low accessibility, the effect of the whip instrument was not significantly influenced by the accessibility degree, while the effects of both the sermon and nudge instruments were, indicating that the combination of behavior public policy instruments and their accessibility degree was more effective in improving the effect of campus policy, with almost equivalent effect.

**Figure 2:**
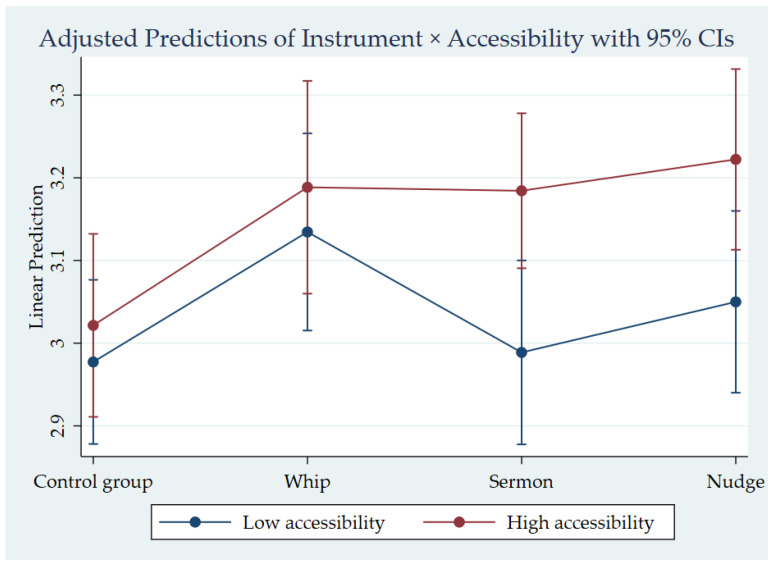
The effectiveness of the policy implementation.

Based on the descriptive statistics and the variance analysis of the experimental data, shown as in Figure 2, it can be concluded that: (1) high policy accessibility is generally superior to low policy accessibility; (2) in high accessibility, the nudge instrument is the most effective type, the whip instrument is the second most effective, and the sermon instrument is the least; (3) in low accessibility, the whip instrument is the most effective, followed by the nudge instrument, with the sermon instrument being the least effective. In fact, the sermon instrument was worse than no policy, and was the least recognized by the target group; it not only failed to realize the expected effect, it had negative effects.

## 6. Conclusions

There is still a lack of relevant research on policy compliance, which is an important part of policy implementation that needs to be verified and supplemented by more scholars’ empirical research. Since the outbreak of COVID-19, how to enhance policy compliance has become particularly urgent and critical, and the introduction of behavioral public policy provides a new perspective for its study. This study examined the behavioral effectiveness of the anti-epidemic polices based on 680 effective samples of college students in China.

The findings of this study are consistent with the findings of Jiangsu, Zhejiang, and Anhui Province, as carried out by Xu Biao et al. [32], and also the national survey made by Dai et al. [33]. The empirical analysis of large samples shows that good policy perception and government trust can help the individual to follow the anti-epidemic measures and achieve the policies’ effects. From foreign examples, citizens in Sweden have high trust in the government. Therefore, the government only urges people to take responsible actions and abide by social distance norms [34], without adopting any traditional punishment policy. From the perspective of the policy intervention mechanism, it was found that the main effects of the Whip and Sermon play a significant role, and the Sermon and Nudge reach a significant level under the high accessibility level. Thus, during the novel coronavirus outbreak, policy makers could consider and compare the effects of different policy instruments—such as the Whip, Sermon, and Nudge instruments—in their decision-making process.

Moreover, in the context of COVID-19, the whip policy instrument has become a more commonly-used tool in the world, such as the fine in France, border control in Australia, the city quarantine in China, and so on. Although this policy instrument can be carried out in a short period of time, a rough policy instrument often leads to public dissatisfaction, which is not a long-term solution. Therefore, it is very critical to select more effective policy instruments. It was found that Whip and Sermon under high accessibility can play a significant role, and the effect was more effective than rough punishment. It can be seen that in the design of public health policies such as anti-epidemic policies, policy makers should consider the advantages of behavioral public policies, such as their low cost, high applicability, easy implementation, and soft governance, but should also pay attention to improving the accessibility of policies, so that the anti-epidemic policies can be implemented and complied with in effective way. Therefore, unlike classical policy instruments, nudge instruments could be better explored as the most effective instrument during the epidemic period, so as to improve the effectiveness of policy implementation and provide better policy guidance against the coronavirus.

However, this paper has its limitations. Due to the impact of the epidemic situation and the limitation of the accessibility of the experimental subjects, the experimental field of this paper was placed in the university scene, and the experimental scene couldn’t be extended more. In addition, the number of experimental subjects was relatively small, and they were concentrated in Guangdong in China, and the statistical effect of some of the conclusions was not high, such that they need to be tested and discussed more carefully under the research framework.

Nevertheless, in the context of the normalization of anti-epidemic polices, this paper empirically tested the factors, intervention strategies, and heterogeneity of COVID-19 compliance behaviors. Moreover, China’s experience in responding to the COVID-19 epidemic has attracted attention from all other countries. Discussing compliance with anti-epidemic policy in China may provide insights to increase the effectiveness of anti-epidemic policies in other countries.

## Figures and Tables

**Table 1:** Eight experimental conditions.

Accessibility Degree	Policy Instruments			
	Whip	Sermon	Nudge	Control Group
High level	H1 (sign via WeChat + major demerit + for disposal according to law)	H2 (sign via WeChat + slogan)	H3 (sign via WeChat + encourages students to participate in the online anti-epidemic action)	H7 (sign via WeChat)
Low level	H4 (publishes only notices + major demerit + for disposal according to law)	H5 (publishes only notices + publishes only notices)	H6 (publishes only notices + publishes only notices)	H8 (publishes only notices)

**Table 2:** Descriptive statistics of the samples.

Variable	Variable Value	Mean	Std. Dev.	Min	Max
sex	1 = male; 0 = female	0.56	0.50	1	2
age	1 = 19 years old; 2 = 20–24 years old; 3 = 25 years old or older;	1.75	0.50	1	3
education	1 = College; 2 = University; 3 = Master or above	2.04	0.43	1	3
political status	1 = CCP member; 0 = Non CCP member	0.11	0.31	0	1
monthly income	1 = 1000 RMB or less; 2 = 1001–3000 RMB; 3 = 3000 RMB or plus	1.49	0.61	1	3
province	1 = Guangdong; 0 = other provinces	0.73	0.45	0	1
living area	1 = city; 0 = village	0.68	0.47	0	1
confirmed cases of COVID-19	1 = 0–20 cases; 2 = 21–80 cases; 3 = 81 cases or more; 4 = no idea	1.91	0.92	1	4
anti-epidemic policy period	1 = 20200109; 2 = 20200119; 3 = 20200120; 4 = 20200123; 5 = 20200124	3.54	1.42	1	5

**Table 3:** Descriptive statistics of the variables.

Aspect	Variable	Variable Type	Mean	Std. Dev.	Min	Max
**Independent Variables**						
Individual psychology	mood instability	continuous variable	2.48	0.85	1.00	5.00
	psychological pressure	continuous variable	3.50	0.51	1.63	5.00
	estimation about COVID-19	ordinal variable	1.83	0.59	1.00	3.00
Individual behavior	level of updates about COVID-19	ordinal variable	1.56	0.62	1.00	3.00
	off-line participations in social activities	nominal variable	0.20	0.40	0.00	1.00
	Any donation during the COVID-19	nominal variable	0.45	0.50	0.00	1.00
Trust in government	trust in central government	continuous variable	4.35	0.73	1.00	5.00
	trust in local government	continuous variable	4.11	0.82	1.00	5.00
**Dependent Variables**						
Behavior effect of policy	epidemic prevention effect	continuous variable	3.09	0.52	1.25	5.00
	university epidemic prevention effect	continuous variable	3.09	0.43	1.00	4.17

Note: estimation about COVID-19: 1 = within 1 month, 2 = 2–3 months, 3 = 3 months or more; level of updates about COVID-19: 1 = non or within 1 h per day, 2 = 1–2 h per day, 3 = 3 h or more per day; off-line participations in social activities: 1 = Yes; 0 = No; any donation during the COVID-19: 1 = Yes; 0 = No.

**Table 4:** Regression results of the effectiveness of the policy implementation.

	(4)	(5)	(6)
	University Policy Effectiveness	University Policy Effectiveness	University Policy Effectiveness
Instrument (Reference Group = control group)			
whip	0.16 ***		0.16 **
	(0.06)		(0.08)
sermon	0.09		0.01
	(0.05)		(0.08)
nudge	0.13 **		0.07
	(0.05)		(0.08)
Accessibility (Reference Group = low accessibility)			
high accessibility		0.12 ***	
		(0.04)	
Interaction Effect (Reference Group = control group × low accessibility)			
control group × high accessibility			0.04
			(0.08)
whip × high accessibility			0.05
			(0.09)
sermon × high accessibility			0.2 ***
			(0.07)
nudge × high accessibility			0.17 **
			(0.08)
_cons	2.94 ***	2.97 ***	2.98 ***
	(0.04)	(0.04)	(0.05)
Observations	680	680	680
R-squared	0.03	0.02	0.03

Note: *** *p* < 0.01, ** *p* < 0.05.

## Appendix A. Cronbach’s Alpha

**Table A1:** Cronbach’s test.

Variable	Items	Average Interitem Covariance	Cronbach’s Alpha
Epidemic prevention effect	4	0.11	0.61
University epidemic prevention effect	4	0.18	0.62

## Appendix B. Balance Check

**Table A2:** Balance Check.

	H1 n = 84	H2 n = 84	H3 n = 75	H4 n = 83	H5 n = 87	H6 n = 88	H7 n = 87	H8 n = 92	*p*
**Demographic Characteristics**									
sex	0.52	0.59	0.56	0.7	0.57	0.56	0.45	0.57	0.161
age	1.77	1.67	1.81	1.8	1.73	1.74	1.76	1.75	0.809
education	2.06	2	2.04	2.06	2.01	2.06	2.06	2.05	0.935
political status	0.08	0.05	0.14	0.13	0.06	0.11	0.14	0.15	0.331
monthly income	1.52	1.48	1.54	1.51	1.42	1.49	1.49	1.42	0.906
province	0.77	0.71	0.69	0.73	0.77	0.8	0.66	0.67	0.274
living area	0.7	0.75	0.65	0.65	0.63	0.72	0.66	0.66	0.750
confirmed cases of COVID-19	1.82	2.01	1.82	1.88	2.08	1.9	2.07	1.74	0.100
anti-epidemic policy period	3.65	3.64	3.54	3.51	3.46	3.57	3.51	3.47	0.982
**Experimental Actions**									
reading time(s)	21.64	23.23	39.49	22.3	25.15	30.06	26.1	20.95	0.053
click count(times)	1.76	1.36	1.88	1.13	1.31	1.52	1.31	1.24	0.548

## Appendix C. Questionnaire

**COVID-19 Social Attitudes Survey**

**Cover page**
The epidemic of COVID-19 has drawn close attention from the whole country. We, a research team from South China Normal University, are conducting a COVID-19 Social Attitude Survey, aiming to conduct scientific research on the relevant issues of the epidemic. Your answers will bring great help to our research. We solemnly promise that the information obtained will only be used for scientific research and analysis of relevant policy suggestions, and the personal information will never be disclosed. Please feel free to fill in.
It will take 10–15 min to fill in this questionnaire. You can also leave your email address at the end, as we will select 100 participants to send health gifts (such as disinfectant, hand sanitizer, etc.). Thank you very much for your support!
**Part One: Basic information**
**1. Your gender?** Male (1) Female (0)
**2. Your age?** 19 years old (1) 20–24 years old (2) 25 years old or older (3)
**3. Your highest education?** College (1) University (2) Master or above (3)
**4. Your current occupation?** Students in School (1) Government/Government Officers/Civil Servants (2) Corporate Staff (Office/Office Staff) (3) Technical personnel (e.g., engineers, IT programmers, etc.) (4) Professional staff (e.g., doctor/lawyer/sports/journalist/teacher) (5) Ordinary workers (e.g., factory workers/manual workers, etc.) (6) Business service workers (e.g., sales staff/shop staff/waiters, etc.) (7) Self-employed Workers/Contractors (8) Freelancer (9) Agriculture, forestry, animal husbandry and fishery workers (10) Retirement (11) No occupation at present (12)

**Part Two: Concern and understanding of the epidemic**
**5. The following description, whether in line with your mood and state of mind?**

	Strongly disagree (1)	Disagree (2)	Sort of (3)	Agree (4)	Strongly agree (5)
Panic					
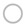
Confused					
Anxious					
Concerned					
Stressed					
Uncomfortable					

**6. What do you think are the main sources of psychological stress for COVID-19?**

	Strongly disagree (1)	Disagree (2)	Sort of (3)	Agree (4)	Strongly agree (5)
Infectivity of virus					
A high mortality rate of patients					
Sequelae after recovery					
Unknown of the illness cause					
No effective treatment					
Extensive news media coverage					
Fear and rumors around					
Mask and notices are seen everywhere					
Gossip from the Internet					

**7. How long do you expect the COVID-19 outbreak to last?**
Within 1 month (1) 2–3 months (2) 3 months or more (3)
**Part Three: Effect of public policy** **8. When did your local government take epidemic control measures?** Around 9 January 2020 (Wuhan reported its first death from pneumonia of unknown cause) (1) Around 19 January 2020 (Important Directive from the Centre on COVID-19) (2) Around 20 January 2020 (Academician Zhong Nanshan confirmed “person-to-person transmission”) (3) Around 23 January 2020 (Wuhan closed the traffic in and out of the city) (4) 24 January 2020 and after (Chinese New Year’s Eve and after) (5)

**9. Please judge the following statements according to your actual feelings.**

	Strongly disagree (1)	Disagree (2)	Sort of (3)	Agree (4)	Strongly agree (5)
The current epidemic prevention measures are very effective					
The current epidemic prevention measures can be implemented to people					
The people around are very conscious of following the current epidemic prevention measures					
At present, the social cost of epidemic prevention measures is very high					
The people around are very supportive of the current prevention measures					
The epidemic prevention and control measures have strengthened my confidence in the country					
I believe the central government can handle the situation well					
I believe the local government can handle the situation well					
The response to the epidemic has boosted my national pride					
As a citizen, I am willing to contribute to my country in its time of need					

**Part Four: Policy compliance experiment materials**
**Intervention Group H1 (Whip × High Accessibility)** Q1 Timing First click (1) Last click (2) Page submit (3) Click count (4)
**Please read the following materials and answer the relevant questions:** (Note: this page will be displayed for **at least 15 s** before jumping to allow you to **fully read** the materials) The outbreak of Novel Coronavirus 2019 (COVID-19) is currently under strain. To reduce the risk of transmission, the Education Department of L Provincial Government issued an urgent notice barring students from returning to university in advance. To respond to the call, Q University announces that **it will punish those who do not follow policy by recording major demerit in the university, and those who seriously violate the policy shall be transferred to the public security for disposal according to law**. Besides, Q University issues the notice through official website and **requires that each student be involved, by forcing each to acknowledge their acceptance of the policy via class WeChat group.**
**Intervention Group H2 (Sermon × High Accessibility)** Q2 Timing First click (1) Last click (2) Page submit (3) Click count (4)
**Please read the following materials and answer the relevant questions:** (Note: this page will be displayed for **at least 15 s** before jumping to allow you to **fully read** the materials) The outbreak of Novel Coronavirus 2019 (COVID-19) is currently under strain. To reduce the risk of transmission, the Education Department of L Provincial Government issued an urgent notice barring students from returning to university in advance. To respond to the call, Q University **edits slogans promoting the measure, such as “anti-epidemic situation, prevention of transmission, less going out, no going back to university”.** Besides, Q University issues the notice through official website and **requires that each student be involved, by forcing each to acknowledge their acceptance of the policy via class WeChat group.**
**Intervention Group H3 (Nudge × High Accessibility)** Q3 Timing First click (1) Last click (2) Page submit (3) Click count (4)
**Please read the following materials and answer the relevant questions:** (Note: this page will be displayed for **at least 15 s** before jumping to allow you to **fully read** the materials) The outbreak of Novel Coronavirus 2019 (COVID-19) is currently under strain. To reduce the risk of transmission, the Education Department of L Provincial Government issued an urgent notice barring students from returning to university in advance. To respond to the call, Q University **encourages students to participate in online anti-epidemic actions, such as posting “I stand for the prevention policy of university Q’ by promising not to go back to university before the situation is controlled.”** Besides, Q University issues the notice through official website and **requires that each student be involved, by forcing each to acknowledge their acceptance of the policy via class WeChat group.**
**Intervention Group H4 (Whip × Low Accessibility)** Q4 Timing First click (1) Last click (2) Page submit (3) Click count (4)
**Please read the following materials and answer the relevant questions:** (Note: this page will be displayed for **at least 15 s** before jumping to allow you to **fully read** the materials) The outbreak of Novel Coronavirus 2019 (COVID-19) is currently under strain. To reduce the risk of transmission, the Education Department of L Provincial Government issued an urgent notice barring students from returning to university in advance. To respond to the call, Q University **encourages students to participate in online anti-epidemic actions, such as posting “I stand for the prevention policy of university Q’ by promising not to go back to university before the situation is controlled.”** Besides, Q University issues the notice through official website, **saying that the requirements will be implemented.**
**Intervention Group H5 (Sermon × Low Accessibility)** Q5 Timing First click (1) Last click (2) Page submit (3) Click count (4)
**Please read the following materials and answer the relevant questions:** (Note: this page will be displayed for **at least 15 s** before jumping to allow you to **fully read** the materials) The outbreak of Novel Coronavirus 2019 (COVID-19) is currently under strain. To reduce the risk of transmission, the Education Department of L Provincial Government issued an urgent notice barring students from returning to university in advance. To respond to the call, Q University **edits slogans promoting the measure, such as “anti-epidemic situation, prevention of transmission, less going out, no going back to university”.** Besides, Q University issues the notice through official website, **saying that the requirements will be implemented.**
**Intervention Group H6 (Nudge × Low Accessibility)** Q6 Timing First click (1) Last click (2) Page submit (3) Click count (4)
**Please read the following materials and answer the relevant questions:** (Note: this page will be displayed for **at least 15 s** before jumping to allow you to **fully read** the materials) The outbreak of Novel Coronavirus 2019 (COVID-19) is currently under strain. To reduce the risk of transmission, the Education Department of L Provincial Government issued an urgent notice barring students from returning to university in advance. To respond to the call, Q University **encourages students to participate in online anti-epidemic actions, such as posting “I stand for the prevention policy of university Q’ by promising not to go back to university before the situation is controlled.”** Besides, Q University issues the notice through official website, **saying that the requirements will be implemented.**
**Intervention Group H7 (Control group × High Accessibility)** Q7 Timing First click (1) Last click (2) Page submit (3) Click count (4)
**Please read the following materials and answer the relevant questions:** (Note: this page will be displayed for **at least 15 s** before jumping to allow you to **fully read** the materials) The outbreak of Novel Coronavirus 2019 (COVID-19) is currently under strain. To reduce the risk of transmission, the Education Department of L Provincial Government issued an urgent notice barring students from returning to university in advance. To respond to the call, Q University issues the notice through official website and **requires that each student be involved, by forcing each to acknowledge their acceptance of the policy via class WeChat group.**
**Intervention Group H8 (Control group × Low Accessibility)** Q8 Timing First click (1) Last click (2) Page submit (3) Click count (4)
**Please read the following materials and answer the relevant questions:** (Note: this page will be displayed for **at least 15 s** before jumping to allow you to **fully read** the materials) The outbreak of Novel Coronavirus 2019 (COVID-19) is currently under strain. To reduce the risk of transmission, the Education Department of L Provincial Government issued an urgent notice barring students from returning to university in advance. To respond to the call, Q University issues the notice through official website, **saying that the requirements will be implemented.**
**Part Five: Manipulation check of H1 & H4**
**10(1). Please read the above material and make your judgment on the following statement.**

	Strongly disagree (1)	Disagree (2)	Sort of (3)	Agree (4)	Strongly agree (5)
The policy of Q University encourages students to cooperate with the prevention and control work by means of mandatory punishment					

**Part Five: Manipulation check of H2 & H5**
**10(1). Please read the above material and make your judgment on the following statement.**

	Strongly disagree (1)	Disagree (2)	Sort of (3)	Agree (4)	Strongly agree (5)
The policy of Q University promotes students to cooperate with the prevention and control work through publicity and education					

**Part Five: Manipulation check of H3 & H6**
**10(1). Please read the above material and make your judgment on the following statement.**

	Strongly disagree (1)	Disagree (2)	Sort of (3)	Agree (4)	Strongly agree (5)
The policy of Q University is to push students to cooperate with the prevention and control work through an imperceptive way					

**Part Five: Manipulation check of H7 & H8**
**10(1). Please read the above material and make your judgment on the following statement.**

	Strongly disagree (1)	Disagree (2)	Sort of (3)	Agree (4)	Strongly agree (5)
The policy of Q university is nothing special					

**Part Five: Manipulation check of H1, H2 & H3**
**10(2). Please read the above material and make your judgment on the following statement.**

	Strongly disagree (1)	Disagree (2)	Sort of (3)	Agree (4)	Strongly agree (5)
Q school’s notification method is quite “down to earth”					

**Part Five: Manipulation check of H4, H5 & H6**
**10(2). Please read the above material and make your judgment on the following statement.**

	Strongly disagree (1)	Disagree (2)	Sort of (3)	Agree (4)	Strongly agree (5)
Q school’s notification method is not quite “down to earth”					

**Part Five: Manipulation check of all**
**10(3). Please read the above material and make your judgment on the following statement.**

	Strongly disagree (1)	Disagree (2)	Sort of (3)	Agree (4)	Strongly agree (5)
I have heard/known about the practice of Q University in similar materials					
I think Q University’s approach is reasonable					

**Part Six: Policy compliance experiment questions**
**11. Please judge the following statements according to your actual feelings.**

	Strongly disagree (1)	Disagree (2)	Sort of (3)	Agree (4)	Strongly agree (5)
I think Q University’s epidemic prevention measures can be implemented to people					
I think the epidemic prevention measures of Q University can achieve the desired effect					
I will conscientiously follow the current epidemic prevention measures of Q University (no student is allowed to return to school early)					
I think Q University needs to spend a lot of manpower and material resources in the process of implementing anti-epidemic measures (no student is allowed to return to school in advance)					
I think the preventive measures adopted by Q University will be widely welcomed by students					
Here is the test question, please select “Agree”					

**Part Six: Basic information**
**12. How long do you follow news about COVID-19 daily?** Non or within 1h per day (1) 1–2 h per day (2) 3 h or more per day (3)
**13. Your monthly income (including salary income, subsidies, investments, part-time jobs, etc.) is about** 1000RMB or less (1) 1001–3000RMB (2) 3000RMB or plus (3)
**14. Your political status?** CCP member (1) Non CCP member (0)
**15. Have you attended off-line participations in social activities in the near 2 weeks?** Yes (1) No (0)
**16. Have you ever made a donation to fight the epidemic?** Yes (1) No (0)
**17. Your current province?** Guangdong (1) Other provinces (0)
**18. Your living area belongs to** City (1) Village (0)
**19. How many confirmed cases with COVID-19 in your city? (Probably)** 0–20 cases (1) 21–80 cases (2) 81 cases or more (3) No idea (4)
Please leave your email address, we will select 100 participants to send health gifts (disinfectant or hand sanitizer) ________________________________________________________________
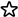

## Appendix D. Multiple Regression Analysis of Variables

**Table A3:** Multiple Regression Analysis of Variables.

	Model	Model	Model
	(1)	(2)	(3)
mood instability	−0.1 ***	−0.1 ***	−0.07 ***
	(0.02)	(0.02)	(0.02)
psychological pressure	0.07	0.06	0.03
	(0.04)	(0.04)	(0.03)
estimation about COVID-19	0	−0.01	0.01
	(0.03)	(0.03)	(0.03)
level of updates about COVID-19		0.01	0.02
		(0.03)	(0.03)
off-line participations in social activities		−0.08 *	−0.06 *
		(0.04)	(0.04)
any donation during COVID-19		0.09 ***	0.07 **
		(0.03)	(0.03)
trust in central government			0.12 ***
			(0.03)
trust in local government			0.15 ***
			(0.03)
demographic variables	controlled	controlled	controlled
_cons	3.25 ***	3.26 ***	1.97 ***
	(0.21)	(0.22)	(0.21)
Observations	645	636	636
R-squared	0.06	0.07	0.26

Robust standard errors are in parentheses; *** *p* < 0.01, ** *p* < 0.05, * *p* < 0.1.
